# BraTS Toolkit: Translating BraTS Brain Tumor Segmentation Algorithms Into Clinical and Scientific Practice

**DOI:** 10.3389/fnins.2020.00125

**Published:** 2020-04-29

**Authors:** Florian Kofler, Christoph Berger, Diana Waldmannstetter, Jana Lipkova, Ivan Ezhov, Giles Tetteh, Jan Kirschke, Claus Zimmer, Benedikt Wiestler, Bjoern H. Menze

**Affiliations:** ^1^Image-Based Biomedical Modeling, Department of Informatics, Technical University of Munich, Munich, Germany; ^2^Department of Neuroradiology, Klinikum rechts der Isar, Munich, Germany

**Keywords:** brain tumor segmentation, anonymization, MRI data preprocessing, medical imaging, brain extraction, BraTS, glioma

## Abstract

Despite great advances in brain tumor segmentation and clear clinical need, translation of state-of-the-art computational methods into clinical routine and scientific practice remains a major challenge. Several factors impede successful implementations, including data standardization and preprocessing. However, these steps are pivotal for the deployment of state-of-the-art image segmentation algorithms. To overcome these issues, we present BraTS Toolkit. BraTS Toolkit is a holistic approach to brain tumor segmentation and consists of three components: First, the BraTS Preprocessor facilitates data standardization and preprocessing for researchers and clinicians alike. It covers the entire image analysis workflow prior to tumor segmentation, from image conversion and registration to brain extraction. Second, BraTS Segmentor enables orchestration of BraTS brain tumor segmentation algorithms for generation of fully-automated segmentations. Finally, Brats Fusionator can combine the resulting candidate segmentations into consensus segmentations using fusion methods such as majority voting and iterative SIMPLE fusion. The capabilities of our tools are illustrated with a practical example to enable easy translation to clinical and scientific practice.

## 1. Introduction

Advances in deep learning have led to unprecedented opportunities for computer-aided image analysis. In image segmentation, the introduction of the U-Net architecture (Ronneberger et al., 2015) and subsequently developed variations like the V-Net (Milletari et al., 2016) or the 3D U-Net (Çiçek et al., 2016) have yielded algorithms for brain tumor segmentation that achieve a performance comparable to experienced human raters (Dvorak and Menze, 2015; Menze et al., 2015a; Bakas et al., 2018). A recent retrospective analysis of a large, multi-center cohort of glioblastoma patients convincingly demonstrated that objective assessment of tumor response via U-Net-based segmentation outperforms the assessment by human readers in terms of predicting patient survival (Kickingereder et al., 2019; Kofler et al., 2019), suggesting a potential benefit of implementing these algorithms into clinical routine.

Recent works present diverse approaches toward brain tumor segmentation and analysis. Jena and Awate (2019) introduced a Deep-Neural-Network for image segmentation with uncertainty estimates based on Bayesian decision theory. Shboul et al. (2019) deployed feature-guided radiomics for glioblastoma segmentation and survival prediction. Jungo et al. (2018) analyzed the impact of inter-rater variability and fusion techniques for ground truth generation on uncertainty estimation. Shah et al. (2018) combined strong and weak supervision in training of their segmentation network to reduce overall supervision cost. Cheplygina et al. (2019) created an overview of Machine Learning methods in medical image analysis employing less or unconventional kinds of supervision.

In earlier years researchers experimented with a variety of approaches to tackle brain tumor segmentation (Prastawa et al., 2003; Menze et al., 2010, 2015b; Geremia et al., 2012), however in recent years the field is increasingly dominated by convolutional neural networks (CNN). This is also reflected in the contributions to the Multimodel Brain Tumor Segmentation Benchmark (BraTS) challenge (Bakas et al., 2018). The BraTS challenge (Menze et al., 2015a; Bakas et al., 2017) was introduced in 2012 at the International Conference on Medical Image Computing and Computer Assisted Intervention (MICCAI), evaluating different algorithms for automated brain tumor segmentation. Therefore, every year the BraTS organizers provide a set of MRI scans, consisting of T1, T1c, T2, and FLAIR images from low- and high-grade glioma patients, coming with the corresponding ground truth segmentations.

Nonetheless, the computational methods presented in the BraTS challenge have not found their way into clinical and scientific practice. While the individual reasons vary, there are some key obstacles that impede the successful implementation of these algorithms. First of all, the availability of data for training, especially of high-quality, well-annotated data, is limited. Additionally, data protection as well as ethical barriers, complicate the development of centralized solutions, making local solutions strongly preferable. Furthermore, there are knowledge and skill barriers, when it comes to the conduction of setting up necessary preprocessing of data, while time and resources are limited.

While individual solutions for several of these problems exist, such as containerization for simplified distribution of code or public datasets, these are oftentimes fragmented and hence difficult to combine. Centralizing these efforts holds promise for making advances in image analysis easily available for broad implementation. Here we introduce three components to tackle these problems. First *BraTS preprocessor* facilitates data standardization and preprocessing for researchers and clinicians alike. Building upon that, varying tumor segmentations can be obtained from multiple algorithms with *BraTS Segmentor*. Finally, *BraTS Fusionator* can fuse these candidate segmentations into consensus segmentations by majority voting and iterative SIMPLE (Langerak et al., 2010) fusion. Together our tools represent *BraTS Toolkit* and enable a holistic approach integrating all the steps necessary for brain tumor image analysis.

## 2. Methods

**Figure 1:**
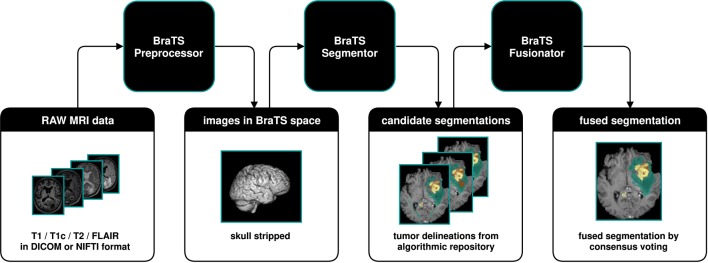
Illustration of a typical dataflow to get from raw MRI scans to segmented brain tumors by combining the three components of the BraTS Toolkit. After preprocessing the raw MRI scans using the BraTS Preprocessor, the data is passed to the BraTS Segmentor, where arbitrary state-of-the-art models from the BraTS algorithmic repository can be used for segmentation. With BraTS Fusionator, multiple candidate segmentations may then be fused to obtain a consensus segmentation. As the Toolkit is designed to be completely modular and with clearly defined interfaces, each component can be replaced with custom solutions if required.

We developed BraTS Toolkit to get from raw DICOM data to fully automatically generate tumor segmentations in NIFTI format. The toolkit consists of three modular components. Figure 1 visualizes how a typical brain tumor segmentation pipeline can be realized using the toolkit. The data is first preprocessed using the BraTS Preprocessor, then candidate segmentations are obtained from the BraTS Segmentor and finally fused via the BraTS Fusionator. Each component can be replaced with custom solutions to account for local requirements^1^. A key design principle of the software is that all data processing happens locally to comply with data privacy and protection regulations.

BraTS Toolkit comes as a python package and can be deployed either via Python or by using the integrated command line interface (CLI). As the software is subject to ongoing development and improvement this work focuses on more abstract descriptions of the software's fundamental design principles. To ease deployment in scientific and clinical practice an up-to-date user guide with installation and usage instructions can be found here: https://neuronflow.github.io/BraTS-Toolkit/.

Users that prefer an easier approach can alternatively use the BraTS Preprocessor's graphical user interface (GUI) to take care of the data preprocessing^2^. The GUI is constantly improved in a close feedback loop with radiologists from the department of Neuroradiology at Klinikum Rechts der Isar (Technical University of Munich) to address the needs of clinical practitioners. Depending on the community's feedback, we plan to additionally provide graphical user interfaces for BraTS Segmentor and BraTS Fusionator in the future. Therefore, BraTS Toolkit features update mechanisms to ensure that users have access to the latest features.

### 2.1. Component One: BraTS Preprocessor

BraTS Preprocessor provides image conversion, registration, and anonymization functionality. The starting point to use BraTS Preprocessor is to have T1, T1c, T2, and FLAIR imaging data in NIFTI format. DICOM files can be converted to NIFTI format using the embedded dcm2niix conversion software (Li et al., 2016).

**Figure 2:**
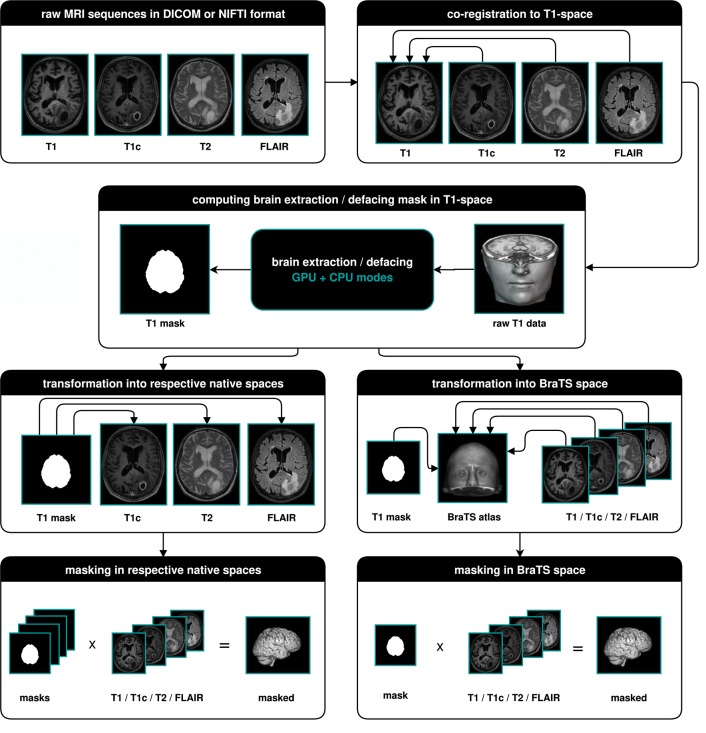
Illustration of the data-processing. We start with a T1, T1c, T2, and FLAIR volume. In a first step we co-register all modalities to the T1 image. Depending on the chosen mode, we then compute the brain segmentation or defacing mask in T1-space. To morph the segmented images in native space, we transform the mask to the respective native spaces and multiply it with the volumes. For obtaining the segmented images in BraTS space, we transform the masks and volumes to the BraTS space using a brain atlas. We then apply the masks to the volumes.

The main output of BraTS Preprocessor consists of the anonymized image data of all four modalities in BraTS space. Moreover, it generates the original input images converted to BraTS space, anonymized data in native space, defacing/skullstripping masks for anonymization, registration matrices to convert between BraTS and native space and overview images of the volumes' slices in png format. Figure 2 depicts the data-processing in detail.

**Figure 3:**
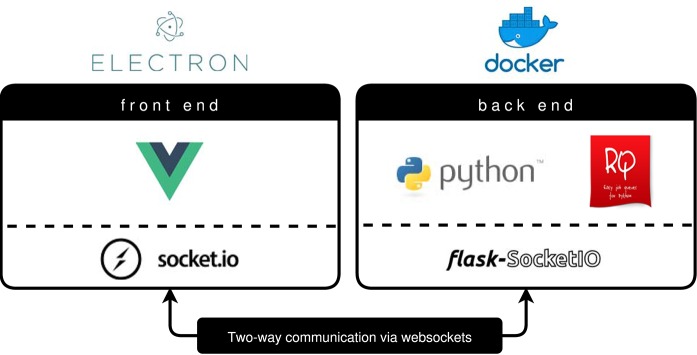
BraTS Preprocessor software architecture (GUI variant). The front end is implemented by a Vue.js web application packaged via Electron.js. To ensure a constant runtime environment the Python based back end resides in a Docker container (Merkel, 2014). Redis Queue allows for load balancing and parallelization of the processing. The architecture enables two-way communication between front end and back end by implementing Socket.IO on the former and Flask-Socket.IO on the latter. In contrast to this the python package's front end is implemented using python-socketio.

BraTS Preprocessor handles standardization and preprocessing of brain MRI data using a classical front- and back end software architecture. Figure 3 illustrates the GUI variant's software architecture, which enables users without programming knowledge to handle MRI data pre-processing steps.

Advanced Normalization Tools (ANTs) (Avants et al., 2011) are deployed for linear registration and transformation of images into BraTS space, independent of the selected mode. In order to achieve proper anonymization of the image data there are four different processing modes to account for different local requirements and hardware configurations:

1. GPU brain-extraction mode
2. CPU brain-extraction mode
3. GPU defacing mode (under development)
4. CPU defacing mode

Brain extraction is implemented by means of HD-BET (Isensee et al., 2019) using GPU or CPU, respectively. HD-BET is a deep learning based brain extraction method, which is trained on glioma patients and therefore particularly well-suited for our task. In case the available RAM is not sufficient the CPU mode automatically falls back to ROBEX (Iglesias et al., 2011). ROBEX is another robust, but slightly less accurate, skull-stripping method that requires less RAM than HD-BET, when running on CPU.

Alternatively, the BraTS Preprocessor features GPU and CPU defacing modes for users who find brain-extraction too destructive. Defacing on the CPU is implemented via Freesurfer's mri-deface (Fischl, 2012), while deep-learning based defacing on the GPU is currently under development.

### 2.2. Component Two: BraTS Segmentor

The Segmentor module provides a standardized control interface for the BraTS algorithmic repository^3^ (Bakas et al., 2018). This repository is a collection of Docker images, each containing a Deep Learning model and accompanying code designed for the BraTS challenge. Each model has a rigidly defined interface to hand data to the model and retrieve segmentation results from the model. This enables the application of state-of-the-art models for brain tumor segmentation on new data without the need to install additional software or to train a model from scratch. However, even though the algorithmic repository provides unified models, it is still up to the interested user to download and run each Docker image individually as well as manage the input and output. This final gap in the pipeline is closed by the Segmentor, which enables less experienced users to download, run and evaluate any model in the BraTS algorithmic repository. It provides a front end to manage all available containers and run them on arbitrary data, as long as the data conforms to the BraTS format. To this end, the Segmentor provides a command line interface to process data with any or all of the available Docker images in the repository while ensuring proper handling of the files. Its modular structure also allows anyone to extend the code, include other Docker containers or include it as a Python package.

### 2.3. Component Three: BraTS Fusionator

The Segmentor module can generate multiple segmentations for a given set of images which usually vary in accuracy and without prior knowledge, a user might be unsure which segmentation is the most accurate. The Fusionator module provides two methods to combine this arbitrary number of segmentation candidates into one final fusion which represents the consensus of all available segmentations. There are two main methods offered: Majority voting and the selective and iterative method for performance level estimation (SIMPLE) proposed by Langerak et al. (2010). Both methods take all available candidate segmentations produced by the algorithms of the repository and combine each label to generate a final fusion. In majority voting, a class is assigned to a given voxel if at least half of the candidate segmentations agree that this voxel is of a certain class. This is repeated for each class to generate the complete segmentation. The SIMPLE fusion works as follows: First, a majority vote fusion with all candidate segmentations is performed. Secondly, each candidate segmentation is compared to the current consensus fusion and the resulting overlap score (a standard DICE measure in the proposed method) is used as a weight for the majority voting. This causes the candidate segmentations with higher estimated accuracy to have a higher influence on the final result. Lastly, each candidate segmentation with an accuracy below a certain threshold is dropped out after each iteration. This iterative process is stopped once the consensus fusion converges. After repeating the processes for each label, a final segmentation is obtained.

## 3. Results

The broad availability of Python, Electron.js, and Docker allows us to support all major operating systems with an easy installation process. Users can choose to process data using the command line (CLI) or through the user friendly graphical user interface (GUI). Depending on the available hardware, multiple threads are run to efficiently use the system's resources.

### 3.1. Practicality in Clinical and Scientific Practice

**Figure 4:**
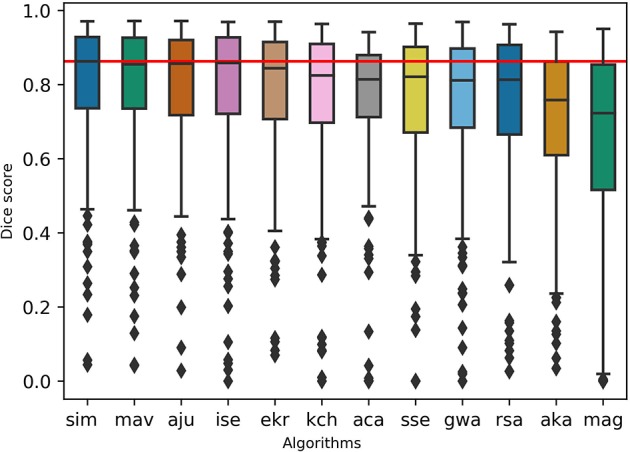
Evaluation of the segmentation results on the BraTS 2016 data set for whole tumor labels on *n* = 191 evaluated test cases. We generated candidate segmentations with ten different algorithms. Segmentation methods are sorted in descending order by mean dice score. The two fusion methods, iterative SIMPLE (sim) and class-wise majority voting displayed on the left, outperformed individual algorithms depicted further right. The red horizontal line shows the SIMPLE median dice score (*M* = 0.863) for better comparison.

**Figure 5:**
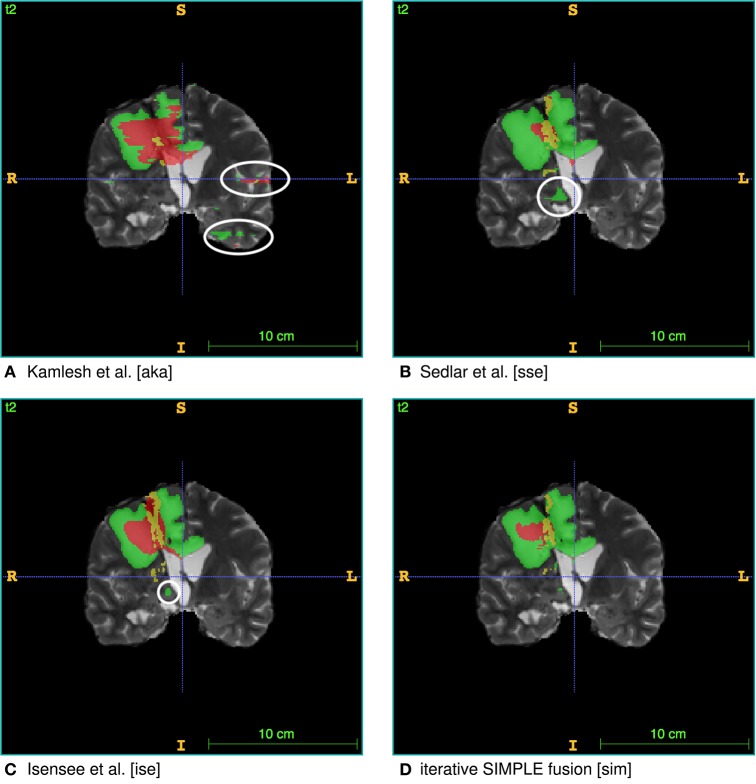
Single algorithm vs. iterative SIMPLE consensus segmentation. T2 scans with segmented labels by exemplary candidate algorithms from **(A)** Pawar et al. (2018), **(B)** Sedlar (2018), and **(C)** Isensee et al. (2017) (Green: edema; Red: necrotic region/non-enhancing tumor; Yellow: enhancing tumor). **(D)** Shows a consensus segmentation obtained using the iterative SIMPLE fusion. Notice the false positives marked with white circles on the candidate segmentations. These outliers are effectively reduced in the fusion segmentation shown in **(D)**.

To test the practicality of BraTS Toolkit we conducted a brain tumor segmentation experiment on 191 patients of the BraTS 2016 dataset. As a first step we generated candidate tumor segmentations. BraTS Segmentor allowed us to rapidly obtain tumor delineations from ten different algorithms of the BraTS algorithmic repository (Bakas et al., 2018). The standardized user interface of BraTS Segmentor abstracts all the required background knowledge regarding docker and the particularities of the algorithms. In the next step we used BraTS Fusionator to fuse the generated segmentations by consensus voting. Figure 4 shows that fusion by iterative SIMPLE and class-wise majority voting had a slight advantage over single algorithms. This effect was particularly driven by removal of false positives as illustrated for an exemplary patient in Figure 5. BraTS Toolkit enabled us to conduct the experiment in a user-friendly way. With only a few lines of Python code we were able to obtain segmentation results in a fully-automated fashion. This impression was confirmed by experiments on further in house data-sets where we also deployed the CLI and GUI variants of all three BraTS Toolkit components with great feedback from clinical and scientific practitioners. Users especially appreciated the increased robustness and precision of consensus segmentations compared to existing single algorithm solutions.

## 4. Discussion

Overall, the BraTS Toolkit is a step toward the democratization of automatic brain tumor segmentation. By lowering resource and knowledge barriers, users can effectively disseminate dockerized brain tumor segmentation algorithms collected through the BraTS challenge. Thus, it makes objective brain tumor volumetry, which has been demonstrated to be superior to traditional image assessment (Kickingereder et al., 2019), readily available for scientific and clinical use.

Currently, BraTS segmentation algorithms and therefore BraTS Segmentor require each of T1, T1c, T2, and FLAIR sequences to be present. In practice, this can become a limiting factor due to errors in data acquisition or incomplete protocols leading to missing modalities. Recent efforts try to bridge this gap by using machine learning techniques to reconstruct missing image modalities (e.g., Dorent et al., 2019; Li et al., 2019).

Other crucial aspects of data preprocessing are the lack of standards for pulse sequences across different scanners and manufacturers, and absence of data acquisition protocols' harmonization in general. For the moment, we address this only with primitive image standardization strategies as described in Figure 2. However, in clinical and scientific practice, we already found our application to be very robust across different data sources. Brain extraction with HD-BET also proved to be sound for patients from multiple institutions with different pathologies (Isensee et al., 2019).

These limitations are in fact some of the key motivations for our initiative. We strive to provide researchers with tools to build comprehensive databases which capture more of the data variability in magnetic resonance imaging. In the longterm this will enable the development of more precise algorithms. With BraTS Toolkit clinicians can actively contribute to this process.

Through well-defined interfaces, the resulting output from our software can be integrated seamlessly with further downstream software to create new scientific and medical applications such as but not limited to, fully-automatic MR reporting^4^ or tumor growth modeling (Ezhov et al., 2019; Lipková et al., 2019). Another promising future direction is to focus on integration with the local PACS to enable streamlined processing of imaging data directly from the radiologist's workplace.

## Data Availability Statement

The datasets generated for this study are available on request to the corresponding author.

## Author Contributions

FK conceptualized the BraTS Toolkit, programmed the BraTS Preprocessor and contributed to paper writing. CB programmed and conceptualized the BraTS Fusionator and BraTS Segmentor and contributed to paper writing. DW, JL, IE, and JK conceptualized the BraTS Preprocessor and contributed to paper writing. GT conceptualized the BraTS Preprocessor software architecture and contributed to paper writing. CZ conceptualized the BraTS Preprocessor and provided feedback on the BraTS Fusionator. BW and BM conceptualized the BraTS Toolkit and contributed to paper writing.

## Conflict of Interest

The authors declare that the research was conducted in the absence of any commercial or financial relationships that could be construed as a potential conflict of interest.

## Footnotes

^1^As an example users who do not want to generate tumor segmentations on their own hardware using the BraTS Segmentor, can alternatively try our experimental web technology based solution nicknamed the Kraken: https://neuronflow.github.io/kraken/.

^2^For an up-to-date installation and user guide please refer to: https://neuronflow.github.io/BraTS-Preprocessor/.

^3^https://github.com/BraTS/Instructions/blob/master/Repository_Links.md#brats-algorithmic-repository

^4^Our Kraken web service can be seen as an an exemplary prototype for this (for the moment it is not for clinical use, but for research and entertainment purposes only). The Kraken is able to send automatically generated segmentation and volumetry reports to the user's email address: https://neuronflow.github.io/kraken/.
